# Neural correlates of intentional switching from ternary to binary meter in a musical hemiola pattern

**DOI:** 10.3389/fpsyg.2014.01257

**Published:** 2014-11-12

**Authors:** Takako Fujioka, Brian C. Fidali, Bernhard Ross

**Affiliations:** ^1^Rotman Research Institute, Baycrest CentreToronto, ON, Canada; ^2^Center for Computer Research in Music and Acoustics, Department of Music, Stanford UniversityStanford, CA, USA; ^3^Brain and Mind Research Institute, Weill Cornell Medical CollegeNew York, NY, USA; ^4^Department of Medical Biophysics, University of TorontoToronto, ON, Canada

**Keywords:** auditory evoked response, MEG, musical meter, timing processing, auditory-motor interaction, anticipatory processing

## Abstract

Musical rhythms are often perceived and interpreted within a metrical framework that integrates timing information hierarchically based on interval ratios. Endogenous timing processes facilitate this metrical integration and allow us using the sensory context for predicting when an expected sensory event will happen (“predictive timing”). Previously, we showed that listening to metronomes and subjectively imagining the two different meters of march and waltz modulated the resulting auditory evoked responses in the temporal lobe and motor-related brain areas such as the motor cortex, basal ganglia, and cerebellum. Here we further explored the intentional transitions between the two metrical contexts, known as hemiola in the Western classical music dating back to the sixteenth century. We examined MEG from 12 musicians while they repeatedly listened to a sequence of 12 unaccented clicks with an interval of 390 ms, and tapped to them with the right hand according to a 3 + 3 + 2 + 2 + 2 hemiola accent pattern. While participants listened to the same metronome sequence and imagined the accents, their pattern of brain responses significantly changed just before the “pivot” point of metric transition from ternary to binary meter. Until 100 ms before the pivot point, brain activities were more similar to those in the simple ternary meter than those in the simple binary meter, but the pattern was reversed afterwards. A similar transition was also observed at the downbeat after the pivot. Brain areas related to the metric transition were identified from source reconstruction of the MEG using a beamformer and included auditory cortices, sensorimotor and premotor cortices, cerebellum, inferior/middle frontal gyrus, parahippocampal gyrus, inferior parietal lobule, cingulate cortex, and precuneus. The results strongly support that predictive timing processes related to auditory-motor, fronto-parietal, and medial limbic systems underlie metrical representation and its transitions.

## Introduction

Listening to the isochronous sound sequence of a metronome beat involves either perceptual grouping or subdividing of the interval at its integer ratios. For example, while listening to an unaccented isochronous pulse, listeners tend to perceive accents every 2nd, 4th, or 3rd stimulus, suggesting that a subconscious grouping of pulses into binary (2 or 4) or ternary (3) sets underlies this perception (Temperley, 1963; Brochard et al., 2003; Abecasis et al., 2005). Similarly, the performance in a tapping task reflects the preference for subdividing the pulse by factors of two or three (Pressing, 1998). Interestingly, the ability to reproduce and temporally rescale even more complex non-isochronous rhythms seems to be further decomposed into sub-units with simple integer ratios such as binary and ternary ratios, which are preferred over non-integer ratios (Collier and Wright, 1995). Furthermore, it appears that the binary ratio is readily preferred over ternary ratio in production and perception (Fraisse, 1956; Povel, 1981; Collier and Wright, 1995), suggesting that the two principles might differ not only in the number of groupings but also in underlying innate mechanisms for timing generation processes (Brochard et al., 2003; Abecasis et al., 2005; Pablos Martin et al., 2007).

**Figure 1:**
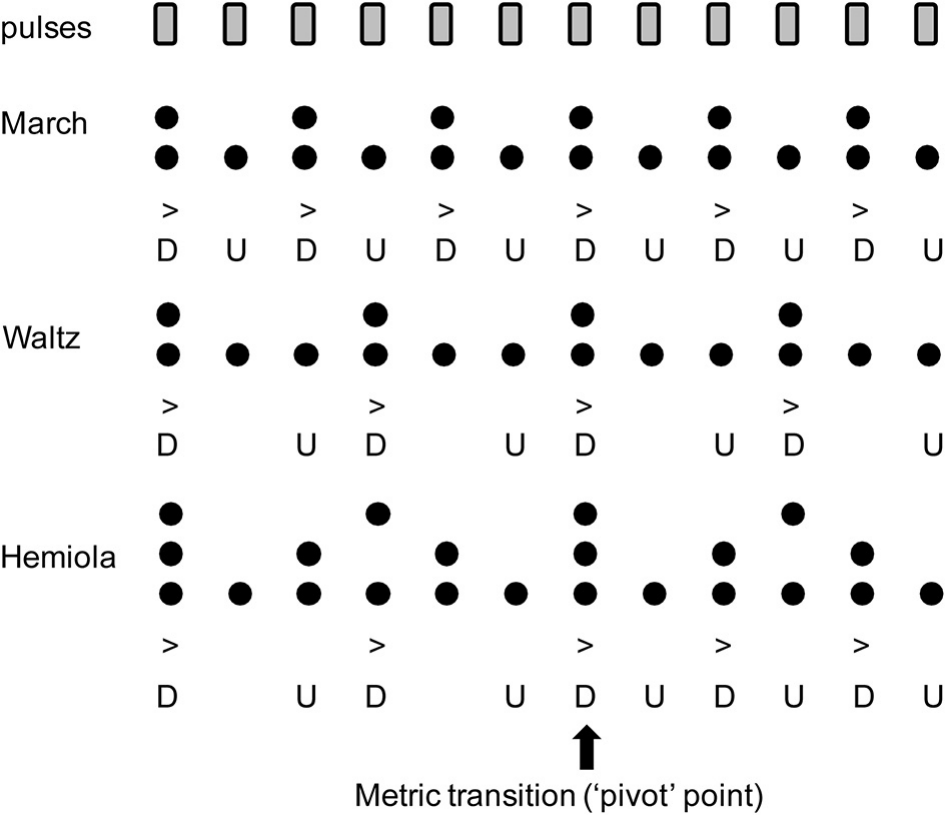
**Accent strength at each pulse position in (Top) binary “march,” (Middle) ternary “waltz,” and (Bottom) “hemiola.”** Note that the upbeat positions (indicated with the letter “U”) have only one layer of metric structures, while the downbeat positions have multiple layers coinciding at the same time. This contributes to the stronger accents in the downbeats (indicated with the letter “D”). In the hemiola pattern, the number of the layers is three due to coexisting binary and ternary meters. At the 1st or 7th position, the strongest accent is derived because of the three coinciding accents. This point also serves as a “pivot” point that allows an intentional switch from ternary to binary meter.

The common perceptual preference for binary and ternary interval ratios is well reflected in the metrical structure of the traditional Western music system. When listening to a musical piece structured within a certain “meter,” listeners perceive subjectively multiple levels of perceptual salience of timing information such as strong or weak accents, similarly as in the illusionary accent described above. In computational music theory, meter can be explained by a collection of grouping levels of the main pulse (“tactus”) which generally falls between 1 and 3 Hz (Large, 2008). Each of the additional pulse levels are subdivisions or higher-level groupings of the tactus in binary and/or ternary ratios (Lerdahl and Jackendoff, 1996; London, 2004). From these pulse levels, a pattern of “strong” and “weak” beats is then derived by points of coincidence shared amongst multiple metric strata, with more points of coincidence conferring higher structural prominence or perceptual “strength” (Figure 1). Thus, in a binary metric cycle (“measure”), the first beat (“downbeat”) is strong, appearing in both lower and higher pulse levels. By contrast, the second beat (“upbeat”) only appears in lower pulse levels, resulting in a weaker perceptual strength (Figure 1, top). A similar schema exists in ternary meter (Figure 1, middle). This pattern extends to higher-order pulse levels, which form the large-scale temporal scaffolding for the entire musical work (Lerdahl and Jackendoff, 1996; London, 2004). The difference in perceptual salience across pulses is considered to form the basis of musical timing expectation (Huron, 2006). Perceptual and motor skills such as auditory memory (Palmer and Krumhansl, 1990; Jones et al., 2002), tapping synchronization (Essens, 1986; Large et al., 2002; Repp, 2007; Repp et al., 2008), auditory discrimination (Pablos Martin et al., 2007; Repp, 2010; Kung et al., 2011), and musical performance (McLaughlin and Boals, 2010) are facilitated by the structure of a metrical hierarchy.

The metric facilitation of auditory and motor tasks predicts underlying “top-down” processing of musical expectations, presumably supported by endogenous neural activity, according to the Dynamic Attending Theory (DAT) (Jones and Boltz, 1989). DAT posits that multiple neural oscillators synchronize with isochronous rhythms, aiding in tracking and anticipation of complex rhythm patterns, such that listeners are able to direct their attention dynamically to a given pulse level, and to musical events at adjacent pulse levels (Large and Kolen, 1994; Large and Jones, 1999; Large, 2000, 2010; McAuley and Jones, 2003). The most important feature of DAT in our context is that the presumed neural oscillators operating simultaneously at several sub-harmonic and harmonic frequencies can successfully reflect a hierarchical structure of musical meter (Large and Jones, 1999; Large and Palmer, 2002). While the DAT and oscillatory models of meter processing have emerged from questions about musical rhythm, they have recently been integrated into wider research questions related to timing processing (Nobre et al., 2007; Kotz and Schwartze, 2010). To adapt to the constantly changing world, the brain seems to infer or predict not only “what” sensory input happens but also “when” it happens to reduce prediction errors and minimize neural resources when things are more predictable (Friston, 2005). These processes, termed “predictive coding” and “predictive timing,” respectively, are thought to be facilitated by endogenous neural oscillatory activities and are crucial for effective perceptual, cognitive and learning processes (Engel et al., 2001). Accordingly, such timing processing in the brain should allow us to expect, perceive, and move in time related to the available cues about musical meter.

Recent electrophysiological studies demonstrate entrainment to hierarchical meter, using auditory evoked responses (AERs) in magnetoencephalography (MEG) and electroencephalography (EEG). When participants imagined higher-level binary and ternary pulse levels during listening to regular pulses, the long-latency AER differentiated between subjective binary and ternary meter conditions as well as between downbeat and upbeat positions in response to a single pulse around after 200 ms toward the next stimulus onset (Fujioka et al., 2010). Using binary, ternary, and quaternary meter conditions, the effects of imagined meter and beat position on the AER were also observed at a similar latency range (Schaefer et al., 2011). Another EEG study with the similar paradigm (Nozaradan et al., 2011) has demonstrated that the AER contained frequency components at the tactus frequency and the additional metric pulse levels (e.g., two or three times slower than the tactus frequency). The latter study by Nozaradan et al. (2011) examined the spectral representation of the AER and found slow oscillatory components matching with the meter frequency, which is complementary to the time domain findings of AER modulation at specific beat position and specific latency in the former two studies (Fujioka et al., 2010; Schaefer et al., 2011). The nature of modulation of the AER in the latency range beyond 200 ms typically relates to higher-level semantic processing of auditory objects. In contrast, shorter latency (<200 ms) responses mainly represent lower-level acoustic information of the stimulus (Ross et al., 2012).

Further EEG studies have also demonstrated expected contrasts between downbeats and upbeats within a given meter during higher-level auditory processing (Brochard et al., 2003; Jongsma et al., 2004; Abecasis et al., 2005; Pablos Martin et al., 2007; Geiser et al., 2009; Potter et al., 2009). The results are in line with enhanced mismatch negativity (MMN) and/or P300 responses, which occur when an anticipated auditory feature corresponding to a metrical downbeat is replaced with another feature, violating the hierarchy of perceptual salience. Given that the generation of MMN and P300 relies on constantly updating the features of the expected events in a memory trace, these results mean that the brain's prediction of “what” seemingly interacts with the salience of the prediction of “when.”

Functional neuroimaging implicated a neural network of both auditory and motor areas functionally associated with rhythm processing and production (Schubotz and Von Cramon, 2001; see Wiener et al., 2011 for meta-analysis). This functional coupling of auditory-motor networks is observed even in perception tasks without a motor component. Specifically, functional magnetic resonance imaging (fMRI) studies concerned with internal encoding of metric information have implicated the basal ganglia (Grahn and Brett, 2007; Grahn and Rowe, 2009), cerebellum (Chen et al., 2008; Bengtsson et al., 2009), and supplementary motor area (SMA) (Chen et al., 2008; Bengtsson et al., 2009), as well as the dorsal premotor cortex and right frontal lobe (Bengtsson et al., 2009). This privileged auditory-motor association in musical rhythm processing has been also found in neural oscillatory activities in the beta-band (13–30 Hz). Beta oscillations are specifically related to the dynamics of the sensorimotor function. Intrinsic beta oscillations are observed in the sensorimotor cortices and motor related brain areas, and their signal power decreases prior and during a movement and increases postmovement (Pfurtscheller and Lopes Da Silva, 1999; Engel and Fries, 2010). In passive listening tasks, endogenous representations of rhythm were found in EEG and MEG (Snyder and Large, 2005; Fujioka et al., 2009, 2012). In these studies, modulations of the beta-band power as well as phase coherence were observed in synchrony with metric pulses.

Moreover, phase coherence in meter-related beta oscillations during auditory-cued finger tapping indicated a network of functional connectivity including auditory and motor cortices as well as basal-ganglia, cerebellum and thalamus (Pollok et al., 2005). While passively listening to isochronous stimuli those areas as well as the anterior cingulate and parahippocampal gyrus were involved in coherent beta oscillation (Fujioka et al., 2012). Although involvement of the parahippocampal gyrus has not been observed in other fMRI findings related to musical rhythm processing (Grahn and Brett, 2007; Chen et al., 2008; Bengtsson et al., 2009; Grahn and Rowe, 2009), a recent fMRI study using naturalistic music stimuli showed association of hippocampus and amygdala with the beat processing (Alluri et al., 2012). Moreover, the parahippocampal gyrus and cingulate were related to acoustic novelty processing reflected in the P300 component in intracranial EEG (Halgren et al., 1995), and MEG (Tarkka et al., 1995) as well as beta-band oscillation in EEG (Haenschel et al., 2000). Thus, the spatial overlap of this beta network with the proposed striato-thalamo-cortical motor network, attention-related fronto-parietal network, and the memory-related limbic network may suggest that shared mechanisms of motor planning and predictive timing exist in meter representation.

In our previous MEG study, we examined AERs of downbeats and upbeats induced by binary and ternary meter tasks (Fujioka et al., 2010). We examined endogenous representations of both binary and ternary meters, to find neural substrates for differential processing of strong and weak metric beats in each meter. Participants alternated between tapping the downbeat (“tap” task, using the right index finger) and imaging each meter over an unaccented, isochronous pulse following auditory cues (“imagine” task). Electromyography (EMG) was used on the right first dorsal interosseous (FDI) muscle to ensure that motor related brain activity did not result from actual motor activity during the imagine tasks and only data from imagine tasks were analyzed. Contrasts between the binary and ternary metric conditions as well as downbeats and upbeats within the waltz condition accounted for the majority of variance in the data. In the contrast between binary and ternary meters, activity was concentrated in the right temporal lobe (including Heschl's gyrus (HG), superior and medial temporal gyri (STG and MTG), and insula) as well as right precentral gyrus and left basal ganglia. In the contrast between downbeat and upbeat AERs within the waltz condition, activations for the downbeat were larger in the left hemispheric basal ganglia and thalamus as well as right temporal regions. These results were consistent with striato-thalamo-cortical network models previously proposed for timing control (Matell and Meck, 2004; Grahn and Brett, 2007; Stevens et al., 2007; Meck et al., 2008; Grahn and Rowe, 2009; Wiener et al., 2011). Additionally, the parahippocampal gyrus was also activated bilaterally in both conditions, although the number and latency of late peaks in the right gyrus varied widely in both contrasts. This seems again in line with the findings about memory processes that modulate the AER as discussed above and might indicate that metric representations are stored and accessed from the hippocampal memory system that is also considered to predict or imagine future events (Martin et al., 2011).

To our knowledge, no research has explored how our brain processes a transition between these two meters, which results in a higher-order complex pattern. From the DAT framework, this is an important question because such transition should presumably require a mental manipulation of switching the timing within a system of neural oscillators. Furthermore, as combination of binary and ternary metric groupings appears to require long-term learning experiences such as exposure over enculturation (Hannon and Trehub, 2005), such voluntary changing between metrical systems can be considered to involve activating endogenous timing processes.

In order to examine neural mechanisms underlying switching between metrical systems, we used the experimental paradigm of alternating between finger-tapping and imagery of metric pattern during isochronous sound sequence, with the 3 + 3 + 2 + 2 + 2 pattern (Figure 1, bottom) as appears in the famous melody of “America” from Leonard Bernstein and Steven Sondheim's *West Side Story*. This pattern, termed “hemiola,” has been featured prominently in Western classical music traditions since the sixteenth century (Russell, 1952; Collins, 1964; Neumann, 1987). In this 12-pulse pattern, the first 6 pulses are grouped in ternary meter, followed by 6 pulses grouped in binary meter. Thus, the metric transition happens between the sixth and seventh tones, which acts as a “pivot,” (Figure 1, bottom). Typically, the hemiola is aimed to cause an interesting rhythmic effect within a musical phrase, syncopating the pulse as the metrical framework transiently shifts between ternary and binary meters. Since this device is used extensively in the musical repertoire, highly trained musicians in Western classical music are well familiar with the hemiola. Accordingly, we examined brain responses only from musicians under the assumption that this familiarity would result in consistent and robust responses, especially since probabilistic learning and musical training have been implicated in strengthening other music-related AERs (Kim et al., 2011). Ternary and binary patterns of the same length used in Fujioka et al. (2010) were also included, allowing direct comparison of AERs between pivot-related down- and up-beats to those in the simple binary and ternary meters.

**Figure 2:**
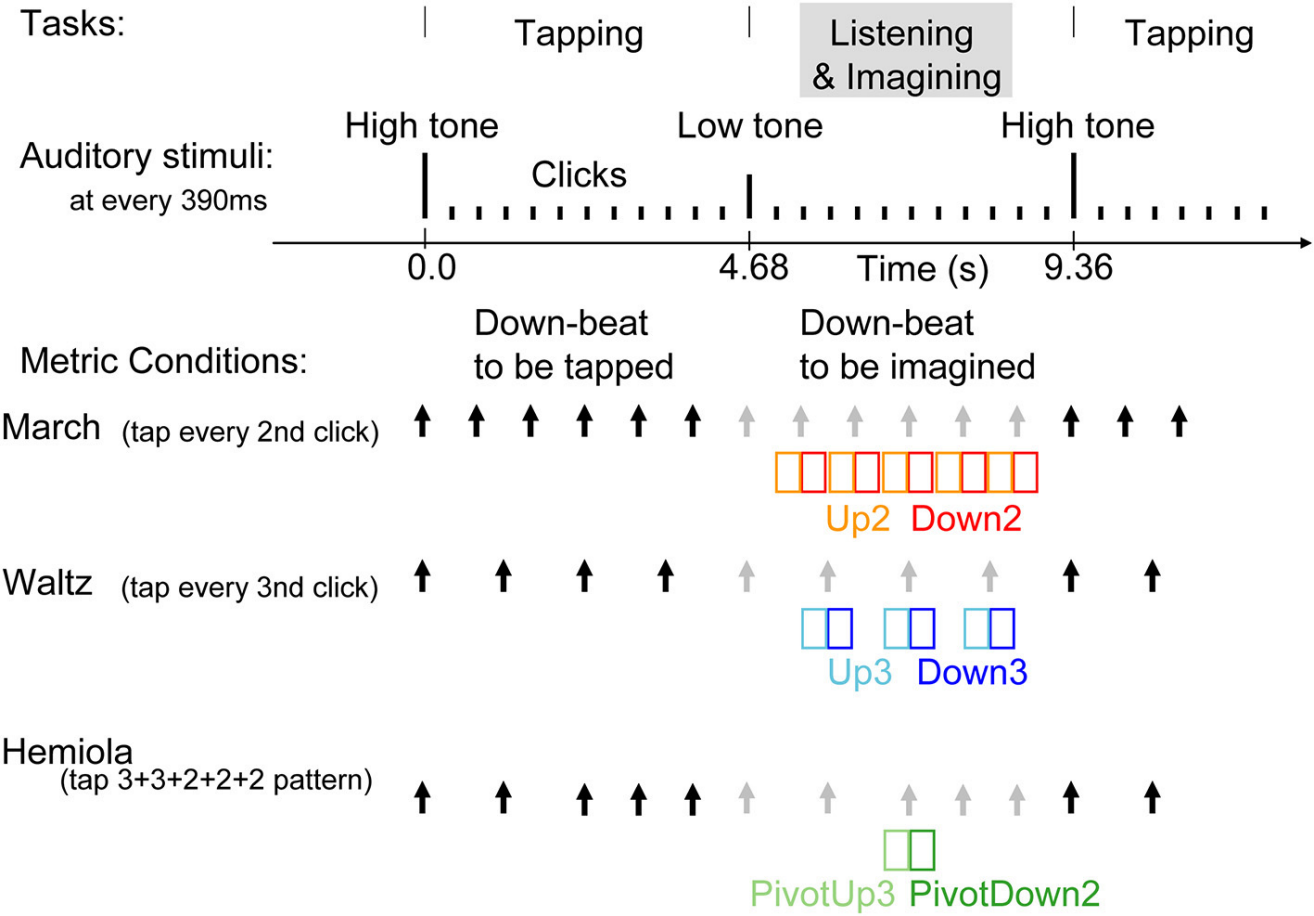
**Stimulus sequence and tasks**. Auditory stimuli were 25 ms tones presented every 390 ms. Changes in pitch cued the beginning and the end of the tapping interval. The same stimulus was used for all three conditions. At a high-pitched 1000-Hz tone, the participants started tapping at every second tone in the march condition, at every third tone in the waltz condition, or twice in every third tone, followed by three times in every second tone, in the hemiola condition. The three conditions were run in separate experimental blocks. The black arrows indicate the downbeats with which the participants' tapping was synchronized in each condition. The gray arrows indicate the imagined, subjectively maintained downbeat positions during the listening interval. In the hemiola condition, the transition of the meter from ternary to binary was termed at “pivot.” We analyzed only the AER in the time window of colored rectangles, namely, upbeats in march and waltz (Up2 and Up3) and the following downbeats respectively (Down2 and Down3), and the upbeat before the pivot point, and the following downbeat (PivotUp3 and PivotDown2).

We examined the MEG in three conditions (Figure 2), namely, march, waltz, and hemiola, to study whether brain activities at downbeat and upbeat positions in the three metric contexts differ. We tested three hypotheses. First, we expected to replicate the binary vs. ternary meter difference in the AER in the auditory cortex (Fujioka et al., 2010). We expected differences in the brain responses between the upbeat position in march (Up2) and waltz (Up3) conditions, and similarly between the downbeat in march (Down2) and waltz (Down3) conditions. Second, during an intentional metric switching, predictive timing processes would be involved for maintaining or updating the internal metric schema, such that the AER to the upbeat just preceding the transition (PivotUp3) would be already different from that to the simple upbeat in waltz (Up3). If the transition starts even earlier, it might already play a role of the upbeat in march (Up2). Moreover, the transition might not be an immediate and complete switch from the up-beat to the down-beat crossing the transition point; these upbeats and downbeats in the hemiola pattern might still carry the representation of both meters, such that simultaneous binary and ternary meter processing gives rise to the underlying polyrhythmic structure as described in Figure 1. Thus, we expected that the brain response at the PivotDown2 position might share homology with those in Down2 and Down3 only partly and/or transiently. Third, the brain areas involved in the meter-related differences would overlap with those found previously in the motor-related areas (Fujioka et al., 2010), regions associated with task switching such as the dorso-lateral prefrontal cortex and dorsal anterior cingulate (Rogers et al., 1998; Macdonald et al., 2000; Johnston et al., 2007; Woodward et al., 2008), and regions of musical and non-musical syntactic processing such as the inferior frontal gyrus (Maess et al., 2001; Koelsch and Mulder, 2002; Tillmann et al., 2006; Koelsch, 2009) due to the intentional effort in changing the metric scheme dynamically.

## Materials and methods

### Participants

Data from 12 healthy, right-handed musicians (9 females; 21–34 years of age, mean of 27 years) were included. Musical qualifications were a minimum of conservatory performance studies or Canadian Royal Conservatory of Music Grade 8 or higher certification in their primary instrument or vocal range. Musicians played a mean of 21 h per week, began musical training at mean age of 7.33 years (range 3–13) and have been studying their instrument a mean of 17.5 years (range 8–28). None had any history of psychological or neurological disorders. The primary and secondary instruments include piano (played by 9 participants), voice (5), guitar (4), woodwinds (4), and strings (3). There were no percussion instrumentalists. The main playing style was classical (10), rock (1), and folk (1). All the musicians had already obtained or been working toward obtaining a music degree/diploma from a postsecondary institution. The study was approved by the Research Ethics Board of the Baycrest Centre for Geriatric Care.

### Stimuli and procedure

Three experimental conditions consisted of binary (“march”), ternary (“waltz”), and a 3 + 3 + 2 + 2 + 2 (“hemiola”) accent pattern (Figure 2). Each condition was tested in separate blocks, each using the same identical sound sequence for stimulation. Within each block, participants alternated repeatedly between blocks of “tap” and “imagine” tasks. They tapped with the right hand index finger on a response key pad. Tap and imagine task stimuli were 12 tones each, consisting of one cue tone followed by 11 click tones in isochronous succession. Clicks were 250-Hz sine tones of 25 ms duration including the rise and fall slopes each 5 ms presented at 390-ms inter-onset intervals (approximately 2.56 Hz). A 1000-Hz cue indicated the beginning of the tapping interval, and a 500-Hz cue the imagine interval. The march block lasted for 190 s (20 tap and imagine cycles), the waltz for 320 s (34 tap and imagine cycles), and the hemiola for 480 s (51 tap and imagine cycles). To obtain an equal number of position-of-interest contrasts in each metric accent structure, four hemiola blocks, two march, and two waltz blocks were used. After practice trials, the order of conditions was counterbalanced across participants, such that the hemiola blocks were always interleaved between march and waltz blocks.

Participants were instructed to tap a rhythmic pattern beginning with the 1000-Hz tap cue. Participants tapped every other beat for the march condition, every three beats in the waltz condition, and in a 3 + 3 + 2 + 2 + 2 tap spacing pattern in the hemiola condition. In each instance, taps coincided with the “downbeat” designation (i.e., Down2 and Down3), with the immediately preceding tone designated as an “upbeat” (i.e., Up2 and Up3). In the hemiola condition, the positions in the pivot transition between march and waltz, were termed as PivotUp3 and PivotDown2.

MEG data were recorded with a 151-channel neuromagnetometer (VSM Medtech) with a continuous sampling rate of 625 Hz for each trial. Participants were seated upright, with their head resting comfortably in the helmet-shaped MEG sensor. Auditory stimuli were presented binaurally via insert earphones E3A (Etymotic Research). Participants were instructed to remain still, avoiding head movement or any extraneous tapping movement during the imagine tasks. Trials were re-run if head movement exceeded a 1-cm threshold or mechanical problems were reported. This happened to 6 trial blocks across two participant sessions, resulting in 16 min extra recording time for both participants. Compliance was monitored by live video. Electromyography (EMG) signals were recorded simultaneously with the neuromagnetic data to monitor tapping motion. Ag/AgCl electrodes were used on both hands, with leads on the FDI muscle and immediately adjacent knuckle. The EMG electrode impedance was kept below 30 kOhms. A ground wire was run from the right collarbone. In addition to EMG data, timing of the key pressing by the right index finger tapping was also recorded with an in-house, custom-made response-button system.

### Data analysis

**Figure 3:**
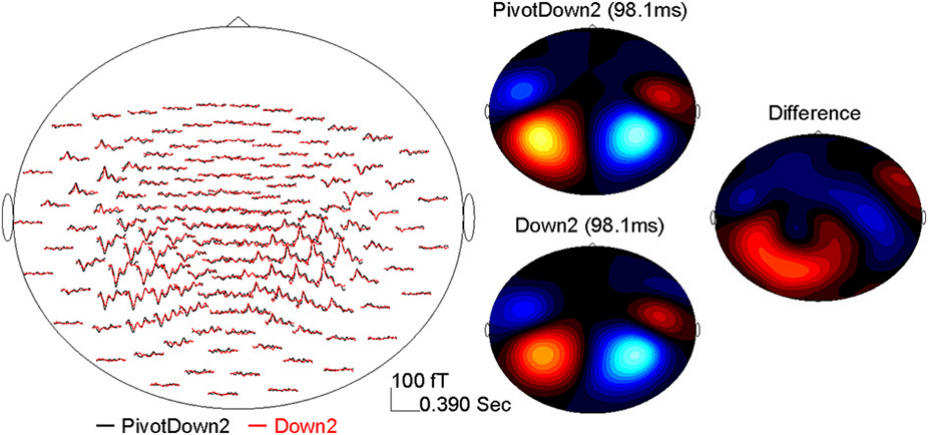
**Example of the AER (0–390 ms) at the MEG sensor level after the down-beat onset in the PivotDown2 in the hemiola and Down2 in the march conditions**. The left panel shows the grand-averaged response in the MEG sensors (top-view). The right panel shows magnetic field topography for each condition at the latency of 98.1 ms, and the difference (PivotDown2-Down2).

The data analysis was focused on AER to stimuli presented during the imagining period (Figure 2). The epoch windows were related to the onsets of stimuli at each position-of-interest (PivotDown2 and PivotUp3 in the hemiola, Down2 and Up2 in the simple march, and Down3 and Up3 in the simple waltz), using a pre-stimulus interval of 400 ms and a poststimulus interval of 800 ms. Sporadic finger movements during this time interval were identified by EMG signals exceeding 25 μV in amplitude or 25 μV/s in its first derivative, after which corresponding MEG epochs were excluded from further analysis. The data were corrected for eye movement artifacts using a principal component analysis (PCA). Any component exceeding 1.5 pT was subtracted from the MEG data. After DC-offset baseline correction using the whole epoch interval, the epochs were averaged for obtaining AERs, which showed magnetic field maps with exclusively bilateral dipolar patterns over the temporal lobes (Figure 3).

**Figure 4:**
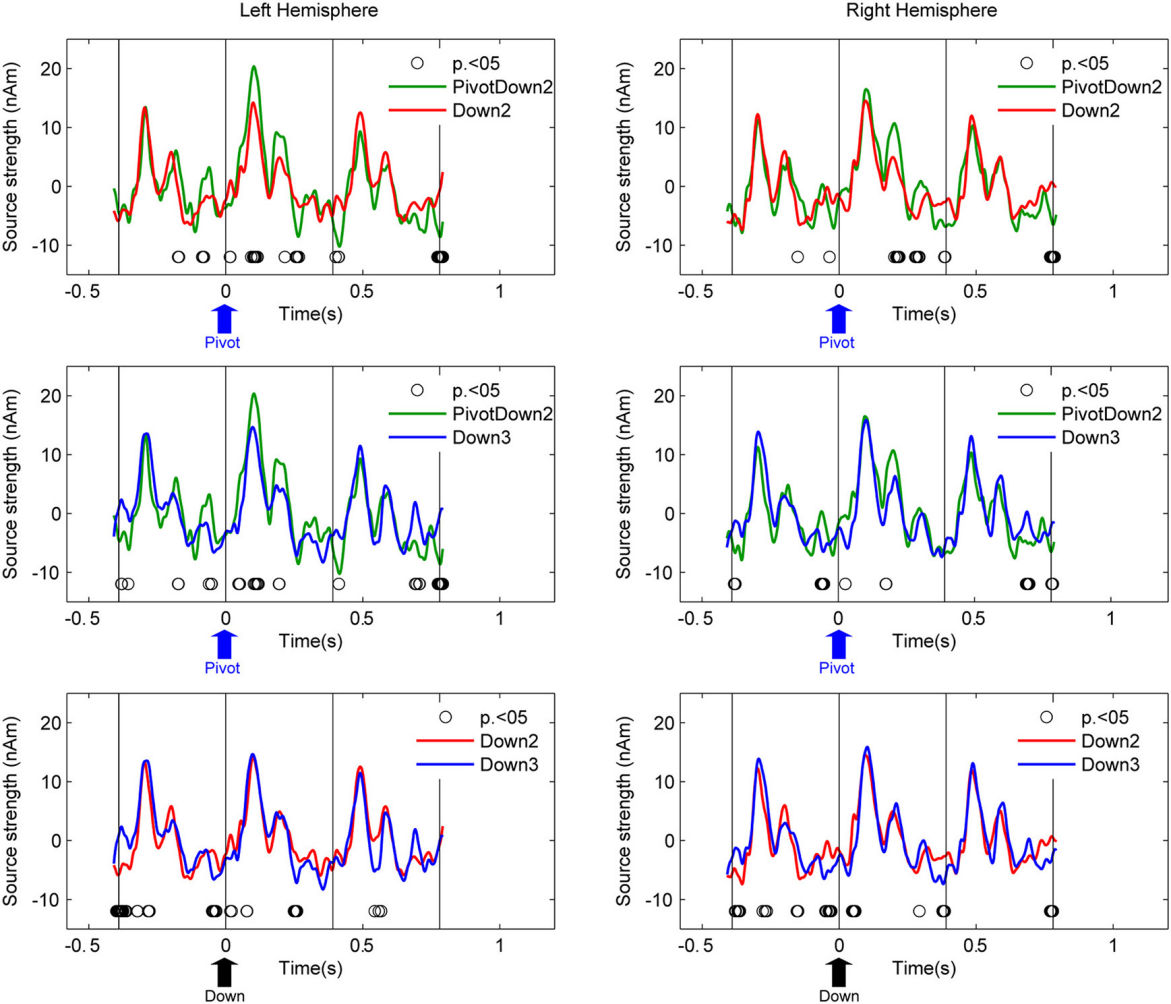
**Grand-average source waveforms of AER from the left and right auditory cortices**. Time zero corresponds to the stimulus onset of the downbeat in the hemiola (PivotDown2), march (Down2) and waltz (Down3) conditions. The vertical lines indicate the stimulus onsets 390 ms before and after the respective downbeat. Thus, the graphs show responses to the downbeat and on preceding and following upbeat. The upward arrow in each plot indicates the time point of pivot position (blue) for PivotDown2, and the downbeat position (black) for Down2 and Down3, respectively. The open circles indicate the time points where the pair-wise comparison using a permutation of the two conditions (hemiola vs. march: top panel, hemiola vs. waltz: middle, and march vs. waltz: bottom) were significant at *p* < 0.05.

Source activities at the left and right auditory cortices were examined with two steps as follows. First, we used equivalent current dipole modeling to estimate the locations and orientations of the dipoles of the AER around the P1m peak with latency of about 90 ms. It has been known that the fast stimulation with 390-ms inter-stimulus interval reduces the N1 response and apparently prolongs the latency of the P1 peak (Näätänen and Picton, 1987). Individual dipole models were calculated as mean across all blocks and conditions. The residual variance for the dipole estimations were 25.3% on average (SD: 11.5). Second, the source activities of the averaged AER were transformed into two source waveforms in the left and right hemispheres respectively for different conditions using source space projection (Tesche et al., 1995; Ross et al., 2000) based on the individual dipole model of each participant. The planned comparison between conditions in a pair-wise manner (PivotDown2 vs. Down2, PivotDown2 vs. Down 3, Down2 vs. Down3, PivotUp3 vs. Up2, PivotUp3 vs. Up3, and Up2 vs. Up3) was performed based on non-parametric permutation tests within each hemisphere (Figure 4). This test used 1000 times permutated data and computed the probability to find the difference in the resampled datasets.

Additionally the whole-brain source activities were estimated using synthetic aperture magnetometry (SAM) (Robinson and Vrba, 1999), a beamformer algorithm that defined a spatial filter on the MEG data in the 0–30 Hz frequency range on a 8 × 8 × 8 mm mesh covering the brain. The SAM approach uses a linearly constrained minimum variance beamformer algorithm (Van Veen et al., 1997; Robinson and Vrba, 1999), normalizes source power across the whole cortical volume (Robinson, 2004), and is capable of identifying activities in auditory (Fujioka et al., 2010, 2012; Ross et al., 2010) and sensorimotor cortices (Jurkiewicz et al., 2006) as well as deep sources such as hippocampus (Riggs et al., 2009), fusiform gyrus, and amygdala (Cornwell et al., 2008). To construct the SAM spatial filter, we used the Montreal Neurological Institute (MNI) template brain, which was derived from averaging the magnetic resonance images (MRI) of 152 brains, based on Talairach standard coordinates as provided in the AFNI software package (Cox, 1996). The source analysis based on individual MRI co-registration with a spherical head model correlates reasonably well with group analysis based on a template brain (Steinstraeter et al., 2009). Thus, the template MRI has been used successfully when individual participant MRIs are not available (Jensen et al., 2005; Fujioka et al., 2010, 2012; Ross et al., 2010). The SAM computation for each condition was based on the single-trial data for each task block (hemiola, march, and waltz) including all down- and up-beats within the block along with the following beat. In the computation of covariance matrices, epoched data was chosen before application of the artifact removal algorithm. This was required to avoid reducing the rank of the covariance matrix when using either PCA or independent component analysis. Subsequently, the spatial filter was applied to the single trials of the evoked magnetic field data obtained under all experimental conditions and calculated the signal power below 30 Hz to create event-related SAMs (ER-SAM; Cheyne et al., 2006). The output measures were time courses of normalized source power for each volume element across the entire time interval.

The obtained four-dimensional ER-SAM maps were down-sampled in time by a factor of eight for data reduction, which resulted in volumetric maps at every 12.8 ms. The voxels, which contained significant activation elicited by the auditory stimuli, were identified by two-sided *t*-tests comparing the mean source power in the first half of the interval and that in the second half by using the data across all conditions. The voxels with *p* < 0.05 were taken into the subsequent partial least square (PLS) analysis. There was no correction for multiple comparisons at this step because statistical inference was made using multivariate analysis described below.

**Figure 5:**
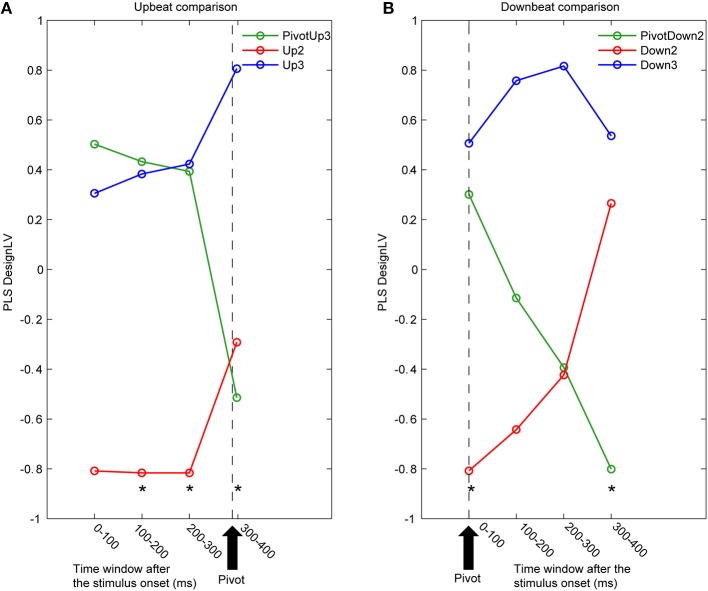
**Time courses of latent variables accounting for the contrast between three metric conditions**. The partial least squares analysis (PLS) was performed for contiguous 100 ms time intervals for **(A)** upbeat positions (PivotUp3, Up2, and Up3) and **(B)** downbeat positions (PivotDown2, Down2, and Down3). Asterisks indicate that the first latent variable was significant by the PLS permutation test (*p* < 0.05).

**Figure 6:**
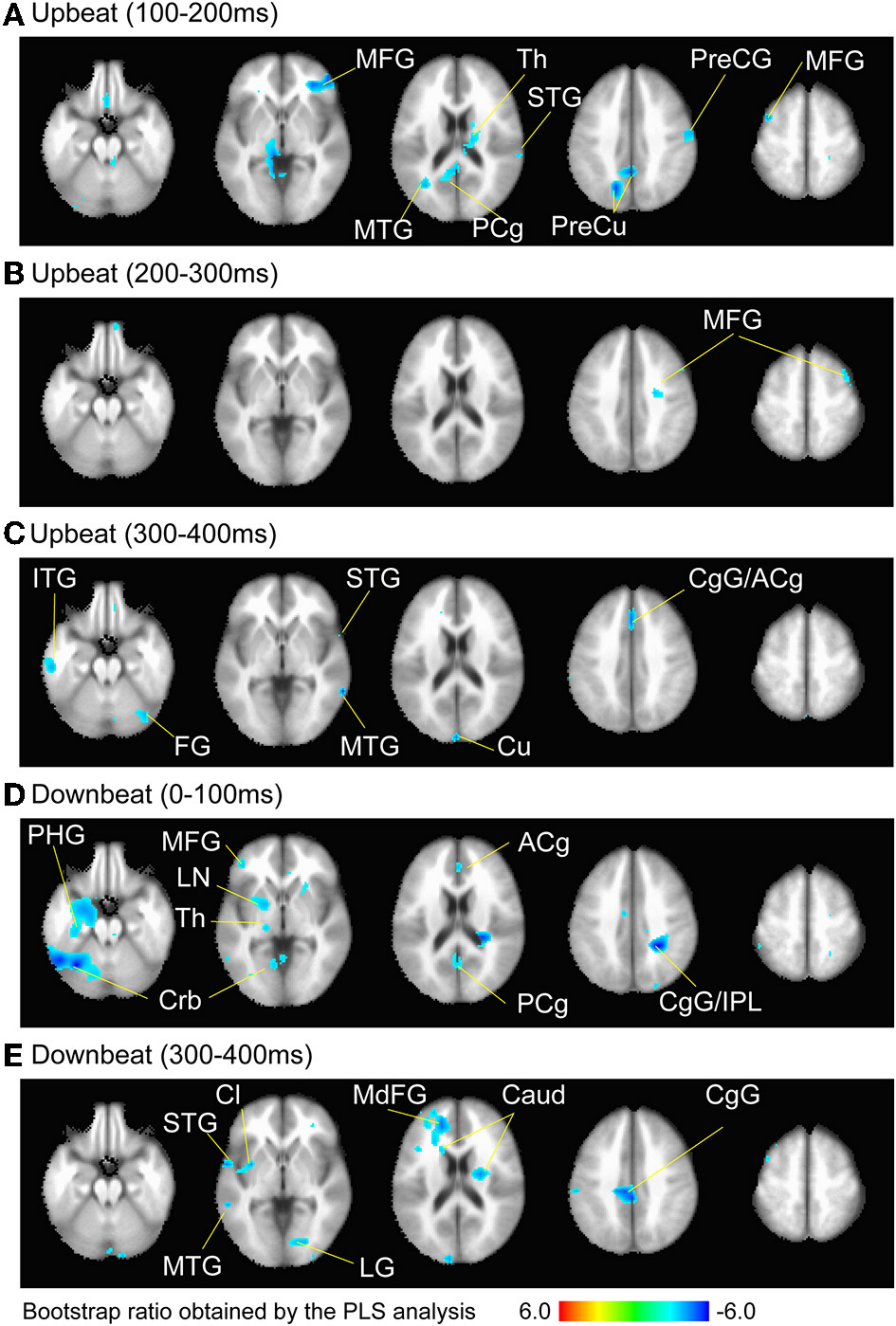
**Brain areas contributing to the contrast between the three metric conditions at the time windows (A) 100–200 ms, (B) 200–300 ms, and (C) 300–400 ms after the upbeat onset, and (D) 0–100 ms and (E) 300–400 ms after the downbeat onset**. The color indicates the bootstrap ratio of a given voxel as the spatial component of the PLS-identified brain-LV. Only the areas where absolute value of the bootstrap score was larger than 3.0, where the contrast was found significant, are shown. Note that thresholding resulted in only cold colored areas correlating with the negative sign of the design-LV values in the contrast pattern indicated in the Figure 5. The anatomical labels are taken from the local maxima and minima of these voxels in Tables 1, 2, respectively. Abbreviation: ACg, anterior cingulate; Caud, caudate; CgG, cingulate gyrus; Cl, claustrum; Crb, cerebellum; Cu, cuneus; FG, fusiform gyrus; IPL, inferior parietal lobule; ITG, inferior temporal gyrus; LG, lingual gyrus; LN, lentiform nucleus; MdFG, medial frontal gyrus; MFG, middle frontal gyrus; MTG, middle temporal gyrus; PCg, posterior cingulate; PHG, parahippocampal gyrus; PreCG, precentral gyrus; PreCu, precuneus; Th, Thalamus; STG, superior temporal gyrus.

A multivariate PLS analysis (McIntosh et al., 1996) was used to examine significant contrasts in spatio–temporal patterns of source activities across different condition. Specifically we performed PLS for the three downbeat conditions (PivotDown2, Down2, and Down3), and for the three upbeat conditions (PivotUp3, and Up2, and Up3) separately for four contiguous time windows every 100 ms after the stimulus onset (0–100, 100–200, 200–300, and 300–400 ms). As a multivariate technique similar to PCA using singular value decomposition (SVD), PLS is suitable to identify the relationship between one set of independent variables (e.g., the experimental design) and a large set of dependent measures (e.g., neuroimaging data). PLS has been successfully applied to time series of multi-electrode event-related potentials (Lobaugh et al., 2001), fMRI data (McIntosh and Lobaugh, 2004; McIntosh et al., 2004) and multi-voxel MEG SAM data (Fujioka et al., 2010). The input of PLS is a cross-block covariance matrix, which is obtained by multiplying the design matrix (an orthonormal set of vectors defining the degrees of freedom in the experimental conditions), and the data matrix (time series of brain activity at each location as columns and participants within each experimental condition as rows). The output of PLS is a set of latent variables (LVs), obtained by SVD applied to the input matrix. Similar to eigenvectors in PCA, LVs account for the covariance of the matrix in decreasing order of magnitude determined by singular values. Each LV explains that a certain pattern of contrast across the experimental conditions (design-LV) (Figure 5) is expressed by a cohesive spatial–temporal pattern of brain activity (brain-LV). This was accompanied by two types of statistical analyses using resampling methods. First, the significance of each LV was determined by a permutation test using 200 times permuted data with conditions randomly reassigned for recomputation of PLS. This yielded the empirical probability for the permuted singular values exceeding the originally observed singular values. An LV was considered to be significant at *p* < 0.05. Second, for each significant LV, the reliability of the corresponding eigenimage of brain activity was assessed by bootstrap estimation using 200 times resampled data with participants randomly replaced for recomputation of PLS, at each time point at each location. The ratio of the activity to its standard error estimated through the bootstrap resampling corresponds to a z-score in parametric testing. This bootstrap ratio indicates statistical significance of the contrast expressed by the LV. Each point in time and space for which the absolute value of the bootstrap ratio was 3.0 (corresponding approximately to *p* < 0.0027) was accepted as significantly contributing to the identified contrast for each LV. For visualization purposes, the significant brain LV was further analyzed to extract the brain activity spatial pattern that was significantly correlated with the design-LV (Figure 6). Among all voxels with a mean absolute value of the bootstrap ratio larger than 3.0 within the time window of the interest, we report the locations of local maxima if the distance between peaks was larger than 20 mm. The Talairach coordinates of the final selection of spatial locations are reported in Tables 1, 2. The Talairach anatomical labels for each location were extracted according to the stereotaxic coordinates from AFNI.

**Table 1:** **Brain locations associated with the upbeat contrast in the time window in the 100–200 ms, 200–300 ms, and 300–400 ms expressed in Talairach brain atlas coordinate system (x:RL[mm], y:AP[mm], z:IS[mm])**.

**Time window**	**Lobe**	**Location**	**Hemisphere**	***x* [mm]**	***y* [mm]**	***z* [mm]**	**Bootstrap ratio**
**(A)**							
100–200 ms	Temporal	Middle temporal gyrus	Right	−71	32	−11	−3.092
	Temporal	Superior temporal gyrus	Left	33	−4	−42	−4.024
	Temporal	Superior temporal gyrus	Left	49	−19	−17	−3.620
	Temporal	Superior temporal gyrus	Right	−64	32	14	−4.216
	Parietal	Inferior parietal lobule	Right	−40	41	29	−3.766
	Parietal	Postcentral gyrus	Left	49	17	30	−3.004
	Parietal	Postcentral gyrus	Right	−24	35	62	−3.677
	Occipital	Lingual gyrus	Left	24	95	−6	−3.304
	Occipital	Middle occipital gyrus	Left	33	64	12	−4.906
	Occipital	Middle occipital gyrus	Right	−48	72	4	−3.662
	Occipital	Precuneus	Left	16	73	44	−5.588
	Occipital	Precuneus	Left	1	49	37	−5.194
	Occipital	Superior occipital gyrus	Right	−32	81	27	−3.326
	Limbic	Thalamus	Right	−16	16	14	−4.357
	Frontal	Inferior frontal gyrus	Right	−48	−49	1	−7.366
	Frontal	Inferior frontal gyrus	Right	−59	−18	1	−3.288
	Frontal	Medial frontal gyrus	Right	−8	−40	25	−5.404
	Frontal	Middle frontal gyrus	Left	40	−7	56	−5.219
	Frontal	Middle frontal gyrus	Left	24	−34	0	−3.627
	Frontal	Middle frontal gyrus	Left	47	−49	−15	−3.159
	Frontal	Middle frontal gyrus	Right	−48	−32	25	−5.12
	Frontal	Precentral gyrus	Right	−48	−1	31	−4.529
	Frontal	Rectal gyrus	Right	−1	−27	−29	−4.170
	Frontal	Superior frontal gyrus	Left	23	−69	15	−3.139
	Frontal	Superior frontal gyrus	Right	−16	−65	10	−3.007
	Cerebellum	Cerebellar tonsil	Left	49	61	−45	−3.302
	Cerebellum	Cerebellar tonsil	Right	−8	54	−37	−3.103
	Cerebellum	Culmen	Left	0	47	−5	−4.892
	Cerebellum	Tuber	Left	33	86	−30	−4.457
	Basal ganglia	Lentiform nucleus	Left	24	−10	−8	−3.062
**(B)**							
200–300 ms	Temporal	Superior temporal gyrus	Left	59	−10	−9	−3.216
	Temporal	Superior temporal gyrus	Left	69	41	5	−3.134
	Temporal	Superior temporal gyrus	Right	−40	57	29	−3.715
	Parietal	Paracentral lobule	Right	−24	42	53	−3.627
	Parietal	Postcentral gyrus	Left	41	43	61	−3.402
	Occipital	Cuneus	Left	1	103	−5	−4.081
	Occipital	Precuneus	Left	41	73	44	−3.485
	Occipital	Precuneus	Left	8	82	51	−3.139
	Limbic	Cingulate gyrus	Left	0	−24	32	−3.44
	Limbic	Cingulate gyrus	Right	−24	0	31	−4.068
	Limbic	Cingulate gyrus	Right	−24	25	30	−3.678
	Frontal	Precentral gyrus	Right	−48	1	55	−3.815
	Frontal	Superior frontal gyrus	Right	−8	−58	−23	−3.46
**(C)**							
300–400 ms	Temporal	Inferior temporal gyrus	Left	57	23	−18	−5.549
	Temporal	Middle temporal gyrus	Left	40	−3	−34	−4.842
	Temporal	Middle temporal gyrus	Left	57	65	28	−4.815
	Temporal	Middle temporal gyrus	Right	−64	48	−3	−5.572
	Temporal	Parahippocampal gyrus	Right	−40	23	−18	−3.373
	Temporal	Superior temporal gyrus	Right	−48	16	6	−3.902
	Temporal	Superior temporal gyrus	Right	−61	−10	0	−3.446
	Parietal	Inferior parietal lobule	Left	65	34	43	−3.645
	Occipital	Cuneus	Left	0	96	19	−4.63
	Occipital	Fusiform gyrus	Right	−32	71	−13	−5.148
	Occipital	Middle occipital gyrus	Left	32	88	11	−3.095
	Occipital	Middle occipital gyrus	Right	−48	80	12	−3.467
	Occipital	Precuneus	Left	0	74	52	−3.885
	Limbic	Anterior cingulate	Left	16	−33	17	−3.374
	Limbic	Thalamus	Left	0	6	−1	−3.337
	Frontal	Inferior frontal gyrus	Right	−32	−11	−17	−3.418
	Frontal	Medial frontal gyrus	Right	0	−29	41	−5.086
	Frontal	Middle frontal gyrus	Left	32	−32	41	−3.183
	Frontal	Middle frontal gyrus	Right	−32	−16	32	−3.216
	Frontal	Middle frontal gyrus	Right	−32	−57	10	−3.103
	Frontal	Precentral gyrus	Left	49	−10	7	−4.314
	Frontal	Precentral gyrus	Left	41	19	69	−3.987
	Frontal	Rectal gyrus	Right	−8	−42	−24	−3.718
	Frontal	Superior frontal gyrus	Left	8	−7	71	−3.193
	Frontal	Superior frontal gyrus	Right	−24	−48	33	−4.317
	Cerebellum	Inferior semi-lunar lobule	Right	−45	77	−43	−3.299
	Cerebellum	Pyramis of vermis	Left	0	70	−29	−3.434

The list indicates the local maxima/minima of the bootstrap ratio obtained (<−3 or >3) by the first latent variable (brain-LV) in the PLS analysis.

**Table 2:** **Brain locations associated with the downbeat contrast in the time window in the 0–100 ms, and 300–400 ms, expressed in Talairach brain atlas coordinate system (x:RL[mm], y:AP[mm], z:IS[mm])**.

**Time window**	**Lobe**	**Location**	**Hemisphere**	***x* [mm]**	***y* [mm]**	***z* [mm]**	**Bootstrap ratio**
**(A)**							
0–100 ms	Temporal	Inferior temporal gyrus	Left	57	55	−4	−3.794
	Temporal	Inferior temporal gyrus	Left	69	23	−19	−3.383
	Temporal	Middle temporal gyrus	Left	48	77	27	−4.141
	Temporal	Middle temporal gyrus	Right	−48	64	4	−3.136
	Temporal	Middle temporal gyrus	Right	−62	31	−10	−3.075
	Temporal	Parahippocampal gyrus	Left	33	29	−19	−3.691
	Temporal	Superior temporal gyrus	Left	33	−4	−42	−5.080
	Parietal	Inferior parietal lobule	Left	53	42	55	−3.462
	Parietal	Supramarginal gyrus	Right	−58	49	29	−3.725
	Occipital	Cuneus	Left	0	95	31	−3.939
	Occipital	Cuneus	Right	−14	88	11	−3.065
	Occipital	Precuneus	Left	0	59	19	−3.504
	Occipital	Precuneus	Right	−27	41	37	−5.859
	Occipital	Precuneus	Right	−28	82	42	−3.523
	Limbic	Anterior cingulate	Right	0	−35	9	−4.135
	Limbic	Anterior cingulate	Right	−9	−17	−9	−3.937
	Limbic	Cingulate gyrus	Left	8	9	46	−3.897
	Limbic	Thalamus	Left	16	24	−1	−3.481
	Frontal	Inferior frontal gyrus	Right	−40	−33	8	−3.671
	Frontal	Inferior frontal gyrus	Right	−63	−17	21	−3.265
	Frontal	Middle frontal gyrus	Left	41	−43	−7	−4.597
	Frontal	Precentral gyrus	Left	8	19	71	−3.594
	Cerebellum	Culmen	Left	9	63	−10	−3.995
	Cerebellum	Culmen	Right	−8	31	−19	−3.417
	Cerebellum	Declive	Left	40	87	−21	−3.111
	Cerebellum	Inferior semi-lunar lobule	Left	17	78	−45	−4.689
	Cerebellum	Tuber	Left	50	62	−28	−6.011
	Cerebellum	Tuber	Right	−39	85	−29	−4.848
	Basal ganglia	Lentiform nucleus	Left	19	−2	−4	−3.912
**(B)**							
300-400ms	Temporal	Middle temporal gyrus	Left	57	40	−3	−4.321
	Temporal	Parahippocampal gyrus	Left	24	48	5	−3.855
	Temporal	Superior temporal gyrus	Right	−56	15	6	−3.907
	Parietal	Paracentral lobule	Left	16	34	53	−3.648
	Parietal	Postcentral gyrus	Left	57	16	14	−4.707
	Parietal	Postcentral gyrus	Left	57	26	38	−3.923
	Occipital	Cuneus	Left	8	96	19	−4.338
	Occipital	Inferior occipital gyrus	Right	−32	87	−13	−5.705
	Occipital	Lingual gyrus	Right	−8	99	−14	−4.159
	Occipital	Middle occipital gyrus	Left	32	95	10	−3.023
	Occipital	Middle occipital gyrus	Right	−48	63	−4	−3.537
	Occipital	Precuneus	Right	−8	66	44	−3.386
	Limbic	Cingulate gyrus	Left	0	25	30	−5.900
	Frontal	Medial frontal gyrus	Left	0	19	69	−3.438
	Frontal	Middle frontal gyrus	Left	41	−7	56	−3.939
	Frontal	Middle frontal gyrus	Right	−32	−41	9	−4.553
	Frontal	Middle frontal gyrus	Right	−48	−16	32	−3.789
	Frontal	Precentral gyrus	Left	57	−9	7	−5.053
	Frontal	Superior frontal gyrus	Left	33	−48	25	−3.777
	Frontal	Superior frontal gyrus	Left	19	−71	10	−3.214
	Frontal	Superior frontal gyrus	Right	−8	−69	−14	−3.639
	Basal ganglia	Caudate	Left	8	−25	8	−5.653
	Basal ganglia	Caudate	Right	−24	8	22	−5.039
	Basal ganglia	Claustrum	Left	33	−2	−1	−4.439

The list indicates the local maxima/minima of the bootstrap ratio (<−3 or >3) obtained by the first latent variable (brain-LV) in the PLS analysis.

## Results

### Auditory evoked responses

AERs were obtained for each condition per participant showing typical dipolar magnetic field patterns in the sensor domain. An example of the AER over a 390-ms beat cycle is shown in the left panel of Figure 3 for the PivotDown2 and Down2 conditions. While response waveforms were generally similar across conditions, multiple differences occurred over the 390-ms beat cycle. The magnetic field of the P1m peak showed dipolar topographies in both hemispheres, while the difference between the two conditions showed a more complex topography. This implies the complexity of the endogenous neural activity overlapping to the exogenous (e.g., stimulus-driven) AER. The auditory cortical source was modeled successfully with a pair of equivalent current dipoles with an upward orientation corresponding to vertex-positive in bilateral temporal lobes for all the participants. The source waveforms based on the individual dipole model exhibited consistent time courses of auditory cortex activity. The first predominant peak around 90–100 ms after the stimulus onset was followed by two smaller positive peaks at approximately 200 and 300 ms latency.

Figure 4 shows the auditory cortex source waveforms and the planned pair-wise comparisons using non-parametric permutation across all the combinations of the conditions separately for upbeats and downbeats. First, the amplitude of the AER in the PivotDown2 in the hemiola condition was larger around the first peak (80–120 ms) and the second peak (200 ms), compared to those to downbeat in march and waltz in the left auditory cortex. In the right auditory cortex, the difference was only significant in the second peak (200 ms). Consistently for both hemispheres, a closer look on the time window 30–50 ms before the downbeat revealed a significant difference between hemiola and simple march and waltz conditions. The bottom panel of Figure 4 shows that for both downbeats and upbeats, march and waltz conditions were significantly different too, but in a distinct time window from those pivot vs. non-pivot comparison.

### Source activities across the whole brain

The first LV consistently explained about 60% of covariance in all eight PLS analyses (61.8, 65.8, 66.0, and 61.9% for the upbeat comparisons, and 55.6, 53.9, 53.8, and 60.9% for downbeat comparisons). Non-parametric permutation tests revealed that the first LV was significant for the upbeat in the 100–200 ms (*p* = 0.0448), 200–300 ms (*p* = 0.0199), and 300–400 ms intervals (*p* = 0.0448), as well as the downbeat 0–100 ms (*p* = 0.0249), and 300–400 ms intervals (*p* = 0.0149). As illustrated in Figure 5, the contrast pattern was expressed dominantly as a shift of the pivot-related brain activity for both upbeat and downbeat comparisons. First, in the upbeat comparison, the brain response at the PivotUp3 resembled that in Up3 in the waltz condition during the first to third time windows, then at the fourth window it became more similar to the Up2 in the march condition (Figure 5A). In the downbeat comparison, this shift occurred earlier; the brain activity at PivotDown2 was in the first time window similar to Down3, but quickly approached Down2 in march condition and completely overlapped Down2 in the third time window (Figure 5B). Notably, in the fourth window, Down2 approached to Down3, but the PivotDown2 stayed separately from either. These contrast patterns illustrate two main findings. First, the overall spatiotemporal patterns of brain activities in march and waltz stayed far apart throughout the time windows across upbeat and downbeat positions. Second, the shift of the brain activity at the hemiola transition point commenced already at the upbeat position and continued after the downbeat.

We examined the brain areas that contributed to transition-related differences expressed in the above described contrast pattern. Figure 6 illustrates the brain areas with the significant first LV for the upbeat (100–200, 200–300, and 300–400 ms) and the downbeat (0–100 and 300–400 ms) comparisons. The focal points spread widely bilaterally including temporal, frontal, parietal lobes and subcortical structures such as the cerebellum and basal ganglia (Tables 1, 2, for upbeat and downbeat comparisons). The areas included superior, middle, and inferior temporal gyri, parahippocampal gyri, precentral and postcentral gyri, superior, middle, and medial frontal gyri, anterior cingulate and cingulate gyri, inferior parietal lobules, caudate, lentiform nucleus, thalamus, and cerebellar regions. Interestingly, only the areas showing a negative bootstrap ratio surpassed the threshold (Figure 6). These are the areas associated with march processing rather than waltz, and consequently, neural processing of the switch from waltz to march in the hemiola condition.

## Discussion

Our present data confirmed that both maintaining a subjective meter as well as intentionally switching the subjective meter context while listening to identical metronome clicks result in significant modulation of spatiotemporal patterns of neural activities expressed as AERs. Note that these responses represent neural activity that is precisely phase-locked to the stimulus onset. Based on well-documented characteristics of the AER waveform related to the stimulus condition, it is no surprise that the temporal patterns of the waveforms in the bilateral auditory cortices were in general very similar across all conditions, as characterized by the prominent positive P1 peak and two subsequent smaller peaks. Despite identical stimuli, significant differences between response waveforms were found, corresponding to the contrasting, endogenous metric contexts. We also identified the brain areas corresponding to the significant contrasts between the conditions, hemiola, and simple march and waltz.

Interestingly, in the auditory cortical source activities the march vs. waltz contrast was more pronounced around the upbeat-associated P1 peak, while hemiola vs. simple meter contrasts were more prominent around the downbeat-associated responses. Since the metric transition occurs in time windows distinct from those differentiating stable march and waltz meters, our data suggest that the switch itself requires recruitment of additional endogenous processes. Additionally, the simple march-waltz contrast shown here replicated and extended findings in previous studies of endogenous metrical hierarchies (Fujioka et al., 2010; Nozaradan et al., 2011). The current results were based on source waveforms that preserved the polarity information of neural activities, a process that was not feasible in previous MEG beamformer (Fujioka et al., 2010) or EEG spectral power analyses (Nozaradan et al., 2011). In the current study, the positive peak responses in the hemiola condition were larger than the simple march and waltz conditions, a finding which might be related to additional computation or inputs to the auditory cortex for establishing a complex metric scheme required for hemiola. Effects of attention and memory related modulation of the AER from frontal, medial, temporal, and parietal lobes have been documented on the components as early as N1 to endogenous component such as P300 (Herrmann and Knight, 2001). In particular, recent research examining temporally-oriented attention showed attenuated N1 and enhanced P2 components when the stimulus was presented at a time that could be predicted from a preceding rhythm (Lange, 2009; Costa-Faidella et al., 2011; Sanabria and Correa, 2013). A similar effect might explain the enhanced positive peaks after the downbeat onset in the hemiola condition, because an attenuated N1 would result in an apparent increase of P1 at the same latency in our data. For the march and waltz conditions without the metric switch, such a strong temporally-oriented attention might not be necessary because of its steady structure.

Most important and novel are our findings about the timing of the AER, which started to differ before the metric transition point, and continued to achieve the shift through the pivot downbeat time window, both shown in the auditory cortical sources (Figure 4) and the whole brain activity pattern (Figure 5). In our PLS results, the brain activity pattern at the pivot upbeat onset was initially almost equivalent to that in the waltz condition, but later became more similar to those in the march condition, before the pivot downbeat onset. From the DAT point of view, the ongoing oscillators to signify each meter should be running in parallel for ternary and binary, but an additional force would be necessary to indicate which meter defines the foreground scheme while the other is kept in the background. In other words, inhibitory mechanisms seem to be necessary to suppress the ongoing ternary oscillator and switch the emphasis onto the binary one. The finding that this shift occurred already before the pivot downbeat strongly supports our general hypothesis that meter-related timing mechanisms are used to predictively orient attention to future events.

A remaining question is how the transition is managed cognitively and what its neural correlates are. On one hand, the participants could perform this task by simply repeating a 12-beat pattern without invoking a march or waltz context instead of consciously switching between meters half way through. Indeed, the literature about behavioral data suggests that people perform initially poorly in the polyrhythmic tapping task but later they learn the task well such that the tapping pattern achieves anticipatory asynchrony typically observed in the simple sensorimotor synchronization task (e.g., one tap for each metronome click) (Tajima and Choshi, 2000). This point of view suggests that our results might be related to the learned motor sequence and its imagery, instead of any timing processing. In fact, even though we have analyzed only the MEG data during the listening task, that segment was always alternated with the production task. Thus, it is possible that our participants may have also additionally covertly rehearsed the motor sequence, resulting in motor related activities captured in our analysis. However, even without any motor imagery, motor-related areas were active in rhythm listening as observed in fMRI (Grahn and Brett, 2007; Chen et al., 2008; Bengtsson et al., 2009; Grahn and Rowe, 2009). Also this explanation cannot entirely account for the reason why the hemiola upbeat or downbeat is differently processed from march or waltz upbeat or downbeats.

As an alternative explanation, we propose that our inner clock system may have the capacity to alternatingly maintain two meters in parallel and the related endogenous activities are captured here. Results from recent fMRI studies using finger tapping tasks corroborate this concept. Compared to simple isorhythmic tapping, performing polyrythmic tapping involves differential activities in the fronto-parietal and motor-related networks (sensorimotor cortex, medial premotor cortex, parietal cortex, basal-ganglia, and cerebellum) (Thaut et al., 2008). Bimanual coordinated tapping on polyrhythm also resulted in the similar brain activity pattern (Ullén et al., 2003). Even if finger tapping was made isochronously, doing so while listening to another rhythm as auditory stimuli recruited fronto-parietal attention network such as inferior frontal gyrus, supramarginal gyrus/inferior parietal lobule (Vuust et al., 2006) and anterior cingulate gyrus (Vuust et al., 2011). The latter two studies offer particularly powerful support to the concept that these areas are involved in maintaining a polyrhythmic context (e.g., multiple meters) in one's mind rather than execution of movements or simple memorization of the pattern itself. Furthermore, fMRI studies demonstrate that during listening to naturalistic music, rhythmic cues are associated with medial temporal lobe and cingulate (Alluri et al., 2012) as well as parietal cortex (Abrams et al., 2013), in addition to the auditory-motor and the frontal-parietal networks. Together with other available evidence, our current results strengthen this working model by showing the metric transition is also processed in these similar neural resources, namely, auditory-motor, frontal-parietal, and medial-limbic networks. Since our task used only a fixed number of cycles to repeat, future research could ask how people may differently perform in voluntary metric switching in an improvizational context, compared with a prescribed one like ours.

Where in the brain are these transitions processed? The significant contrast between march and waltz is shown in the auditory-motor brain areas especially in the time windows where the hemiola metric transition was not yet to occur (100–200 ms in the upbeat comparison, 0–100 ms in the downbeat comparison; Table 1A and Figure 6A, Table 2A and Figure 6D, respectively). Thereafter, frontal and parietal lobes and cingulate cortex contributed significantly to the brain activity patterns where the transition modulated the brain activities, as expressed by the shift of the hemiola condition from waltz to march in the upbeat, and downbeat position (Table 1C and Figure 6C, Table 2B and Figure 6E, respectively). This is in line with our prediction that metric switching demands would resemble task switching in executive functions which typically engaging these areas. Interestingly, this may also be related to a recent fMRI finding which demonstrated the involvement of frontal-parietal and temporal-parietal axes such as medial frontal cortex and precuneus in the higher-order language information processing (e.g., a longer temporal structure integration such as at paragraph level, as opposed to sentence or word level) (Lerner et al., 2011). However, while similar hierarchical complexity and a longer temporal integration demands also occur in the hemiola pattern, the long stimuli duration of the spoken story (tens of seconds) and complex phonetic meter used in their study might recruit additional or different cognitive processes than pure ‘temporal information.’ Still, when listening to spoken language or music, anticipatory processes are most likely in operation. It would be of interest to examine how musical metric-related processes, which require these executive functions and timing processing are different from language-related anticipatory processes.

### Conflict of interest statement

The authors declare that the research was conducted in the absence of any commercial or financial relationships that could be construed as a potential conflict of interest.
